# Radiotherapy Exposure in Cancer Patients and Subsequent Risk of Stroke: A Systematic Review and Meta-Analysis

**DOI:** 10.3389/fneur.2019.00233

**Published:** 2019-03-15

**Authors:** Ruixue Huang, Yao Zhou, Sai Hu, Guofeng Ren, Fengmei Cui, Ping-Kun Zhou

**Affiliations:** ^1^Department of Occupational and Environmental Health, Xiangya School of Public Health, Central South University, Changsha, China; ^2^Institute for Environmental Medicine and Radiation Health, The College of Public Health, University of South China, Hengyang, China; ^3^Beijing Key Laboratory for Radiobiology, Department of Radiation Biology, Beijing Institute of Radiation Medicine, Academy of Military Medical Sciences, Beijing, China; ^4^Department of Nutrition and Food Hygiene, Xiangya School of Public Health, Central South University, Changsha, China; ^5^Department of Radiation Medicine, School of Radiation Medicine and Protection, Medical College of Soochow University, Suzhou, China

**Keywords:** cancer, stroke, relative risk, meta-analysis, radiotherapy

## Abstract

**Background:** Cancer patients who have undergone radiotherapy may have an increased risk of subsequent stroke. A clear and detailed understanding of this risk has not been established.

**Methods:** A search for research articles published from January 1990 to November 2017 in the English language was conducted. Subsequent stroke risk in cancer survivors was compared using relative risk (RR) and 95% confidence intervals (CI) according to whether or not radiotherapy was given.

**Results:** A total of 12 eligible studies were identified including 57,881 total patients. All studies were retrospective, as no prospective studies were identified. The meta-analysis revealed a higher overall risk of subsequent stroke in cancer survivors/patients given radiotherapy compared to those not given radiotherapy (RR: 2.09, 95% CI: 1.45, 3.16). In addition, compared to patients not given radiotherapy, there was an increased risk of subsequent stroke for radiotherapy treated patients with Hodgkin's lymphoma (RR: 2.81, 95% CI: 0.69, 4.93) or head/neck/brain/nasopharyngeal cancer (RR: 2.16, 95% CI: 1.16, 3.16), for patients younger than 40 years (RR: 3.53, 95% CI: 2.51, 4.97) or aged 40–49 years (RR: 1.23, 95% CI: 1.09, 1.45) and for patients treated in Asia (RR: 1.88, 95% CI: 1.48, 2.29), the United States (RR: 1.62, 95% CI: 1.01, 2.23), or in Europe (RR: 4.11, 95% CI 2.62, 6.45).

**Conclusions:** The available literature indicates an approximate overall doubling of the subsequent stroke risk in cancer patients given radiotherapy. The elevated risk was generally statistically significant according to cancer type, baseline patient age and region or country where treatment was given. Caution is required in interpreting these findings due to the heterogeneity of populations represented and lack of standardization and completeness across published studies. Further, if real, we cannot conclude the extent to which patient, treatment and/or investigational factors are responsible for this apparent elevated risk. An objective and more detailed understanding of the risks of radiotherapy, and how to prevent them, is urgently required. It is the responsibility of all who provide cancer services to ensure that the experience of all their patients is documented and analyzed using quality registries.

## Introduction

World-wide arterial disease (including stroke) and cancer are the leading causes of death [see World Health Organization website: http://www.who.int/gho/ncd/en/ (1–4)]. Concerns regarding the risk of subsequent stroke after radiotherapy for cancer is rising as evidence grows regarding how ionizing radiation from radiotherapy damages the heart and cerebral vessels (5). However, it remains unclear whether radiotherapy increases subsequent stroke risk in cancer patients compared to cancer patients given other or no specific treatment. Some studies have indicated an increased stroke rate with radiotherapy (6) and others have not (7, 8).

Possible reasons why the previous studies have varied with respect to stroke risk with radiotherapy include confounding factors. For instance, radiation dose and age at first exposure may affect stroke risk. Stroke risk may be different according to countries or region or may vary according to different cancer types. Therefore, we conducted a meta-analysis of subsequent stroke rate in cancer patients according to whether or not they received radiotherapy. Further, we performed meta-analyses according to cancer type, baseline patient age and region where the radiotherapy was given.

## Methods

This was a meta-analysis of radiotherapy cancer patients vs. non-radiotherapy cancer patients and by subgroups according cancer type, baseline patient age, and region where the treatment was given. Literatures published from January 1990 to November 2017 were considered. Stroke incidence was compared between cancer patients given any radiotherapy exposure and those not given any radiotherapy exposure. Baseline patient age was divided into 4 ranges, <40, 40–49, 50–59, >60 years. We also collected information on radiotherapy dose in each eligible study.

### Study Selection

#### Inclusion Criteria

Studies were included in the meta-analysis if they: evaluated radiotherapy-treated cancer patients, including any type of cancer patients; included a control group who received non-radiotherapy treatments such as surgery or chemotherapy; utilized any dose and radiation type involving radionuclide decay (e.g., gamma rays) or machine-produced beams (e.g., X-rays and electron beams); the exposure of interest was radiotherapy for cancer patients, the outcome was stroke, and the studies reported relative risk (RR) or hazards ratio (HR) values with 95% CIs. RR is a measurement of relative differences. An average annual rate of cancer in radiotherapy and non-radiotherapy patients was also calculated. We included case-control, cohort studies, and randomized trials. Only English language studies were included. We included studies with any definition of stroke, including due to cerebral ischemia or hemorrhage, with or without systematic brain imaging and no matter the duration of the neurological deficit (< or >24 h). Only the first stroke/patient after radiotherapy was used in the analyses.

#### Exclusion Criteria

Studies were excluded from the meta-analysis if they did not involve a cohort of cancer patients or did not discuss radiotherapy treatments. Publication types comprising letters, correspondence articles, case reports, and conference abstracts were also excluded.

### Search Strategy

Two independent staff members searched academic databases for records dating from January 1990 to November 2017. Electronic databases were used: PubMed, SpringerLink, Embase, Cochrane Library, Elsevier/ScienceDirect, Medline, Orbis, and Web of Science. The following search terms were used: (“stroke” [MeSH Terms] OR “stroke” [All Fields]) AND (“neoplasms” [MeSH Terms] OR “neoplasms” [All Fields] OR “cancer” [All Fields]) AND (“cohort studies” [MeSH Terms] OR (“cohort” [All Fields] AND “studies” [All Fields]) OR (“cohort studies” [All Fields] OR “cohort” [All Fields]) OR (“ionizing” [All Fields]) OR (“radiation” [All Fields]) OR “radiotherapy” [All Fields]). Article references were examined for additional studies that may have been missed in the initial search.

### Data Collection

Two independent staff members collected the relevant data from each study, including: first author name, year of publication, publication country, cohort follow-up duration, number of participants, baseline age, number of stroke cases, range of radiation dose (highest, lowest, fractional, and median and/or average total dose), adjusted and unadjusted RR (95% CI) for stroke and confounders. Adjusted RR was used in the analyses when available. Unadjusted RR was used in the analyses only when adjusted RR was not published. When a RR for a particular cancer subtype was not published, we used the RR from the whole sample of cancer patients that included patients with that particular cancer subtype, according to whether or not they were given radiotherapy. Study eligibility was confirmed when both reviewers reached consensus on inclusion (Table 1). If any required information was not available in the published article, the authors were contacted (at the email address provided in the article) for additional information.

**Table 1:** Characteristics of included studies.

**Study**	**Total patients**	**Baseline** **age (Years)**	**Follow-up (Years)**	**CA type (Patient no.)**	**Radiation dose**	**No. of “Stroke” cases given or not given RT**	**Average annual “Stroke” rate ± RT**	**Adjusted and/or unadjusted relative risk of “Stroke” and 95% CI with RT compared to without RT and adjusted factors**
1. De Bruin et al. (6): Netherlands	2,201	Median: 52	Mean: 17.5	HL (2,201)	Highest: 66 Gy and lowest 30–40 Gy	RT:21/609 Non RT: 1/1,592	RT: 0.27 Non-RT: 0.12	Adjusted RR: **2.5** (1.1, 5.6)^▴^^$^^∧^ (adjusted for baseline age)
2. Arthurs et al. (9): Canada	14,069	Range: 35–75	Mean: 11	Head and neck (14,069)	N/P	RT:479/10949 Non-RT:152/3120	RT: 0.21 No-RT: 0.24	Unadjusted Overall RR: **1.70** (1.41–2.05) Adjusted Overall RR:**1.70** (1.41–2.04)^▴^^$^^∧^ (adjusted for baseline age, comorbidity status)
3. Huang et al. (10): Taiwan	10,172	Mean: 53 <45:2,873; 45–54: 3,414; 55–64:2134; 65–74:1293; >75: 458	Mean: 5.8	Oral (6,124) Oropharynx (511) Hypopharynx (801) Nasopharyngeal (2,105) Nasal cavity (226) Salivary gland (352) Other (53)	N/P	RT:167/5781 Non-RT:126/4391 (Not published by CA type)	RT: 0.655 No-RT: 0.748 (Not published by CA type)	Overall unadjusted RR: **1.13** (0.9, 5.1)^▴^^$^^∧^ Adjusted RR in patients aged <55 y: **1.76** (1.22–2.56)^!^ Adjusted RR in patients aged >55 y: **0.74** (0.54–1.02)^!^ (Both RRs adjusted for co-morbidities, geographic region, urbanization, socio-economic status)
4. Hayes et al. (11): USA	413	<80	Range: 0.17–12.17	Head and neck (413)	MD: 64.14 Gy Range: 40–80 Gy	RT:20/291 Non RT: 9/122	N/P	Adjusted RR: **2.09** (1.28–3.22)^▴^^$^^∧^ (adjusted for sex, age)
5. Van Dijk et al. (12): Netherlands	1,360	Range: <18	Mean: 24.9	Brain (109) Leukemia/ Lymphoma (592) Soft tissue sarcoma/ Kidney/Bone/ Neuroblastoma/ Retinoblastoma/ Thyroid/ Hepatoblastom (659)	Cranial radiation: median 39.2 Gy, range: 22.3–76.7 Gy Brain CA: MD 24.8 Gy, range:22.3–76.6 Gy Supra-diaphragmatic radiation therapy MD:33.2 Gy,range: 15.0–41.1 Gy Neck CA: MD 38.6 Gy, range:37.3–40.0 Gy Thorax: MD 25 Gy, range:15.0–31.0 Gy Spine: MD 34.6 Gy, range:31.1–41.1 Gy	RT:28/672 No-RT:5/688; brain (9), leukemia (6) lymphoma (10)/ malignant (1) histiocytoma(1) soft tissue sarcoma (1) kidney tumor(1)	RT: 0.082 No-RT:0.023 (Not published by CA type)	Adjusted Overall RR: **8.15** (2.85–23.3) ^▴^^$^ (Not published by CA type)
6. Mueller et al. (13): USA	18,204	Range: <20	Mean: 23.3	Brain (1,810) Leukemia (4,763) Neuroblastoma (94) Soft tissue sarcoma (1,241) Kidney (1,250) Bone (1,183) HL (1,925) NHL (1,068)	Brain dosage categories: 50+Gy, 30–49 Gy, 1.5–29 Gy	RT:292 14186 Non-RT:17/4018 Brain: 125 Leukemia: 71 Neuroblastoma: 5 Soft tissue sarcoma:18 Kidney:6 BoneCA:14 HL:44 NHL: 9	RT: 0.088 No-RT: 0.018 (Not published by CA type)	Overall Adjusted RR: **7.8** (4.7–13.0)^▴^^∧^ Brain: Adjusted RR **30.1** (17.9–50.8)^$^ Leukemia: Adjusted RR **8.2** (4.6–14.5) Neuroblastoma: Adjusted RR **5.2** (1.1–24.24) Soft tissue sarcoma: Adjusted RR**4.6** (2.3–9.2) Kidney: Adjusted RR**3.3** (0.8–13.3) Bone: Adjusted RR **2.8** (1.3–5.8) HL: Adjusted RR **4.4** (2.5–7.8)^$^ NHL: Adjusted RR **42.6** (1.2–5.9) (All RRs adjusted for baseline age)
7. Hung et al. (14): Taiwan	560	≤65 and >65	Mean: 2	Lung (560)	TD:64.8 Gy vs. 45Gy	RT:5/448 non-RT:7/112	RT: 1.60 No-RT: 0.66	Unadjusted RR: **3.28** (1.15–9.37) (NP) Adjusted RR: **4.19** (1.44–12.22) (NP)^▴^^$^^∧^ (adjusted for baseline age, diabetes mellitus)
8. Donnellan et al. (15): USA	172	Mean: 63	N/P	Thoracic (172) Breast (51) Lung (5) HL (94) NHL (10) Others (12)	Thoracic: N/P Breast CA (TDrange:50–6 0Gy) Lung CA (TD:60 Gy) HL (TDrange:40–45 Gy) NHL (TDrange: 40–45 Gy) Others(TD range: 40–45 Gy)	RT: 14/172 No-RT: 15/172 (Not published by CA type)	RT: 0.904 No-RT:0.968 (Not published by CA type)	Unadjusted Overall RR: **1.08** (0.55, 2.13) ^▴^^$^^∧^ (Not published by CA type)
9. Van den Belt-Dusebout et al. (16): Netherlands	2,512	Median: 38.3	Median: 18.4	Testicular CA (2,512)	N/P	RT: 27/1116 Non RT:14/1223	RT: 0.097 Non-RT:0.023	Adjusted RR: **1.2** (0.8–1.7)^▴^^∧^ (adjusted for sex, age
10. Chu et al. (17): Taiwan	4,615	Mean: 51.16 <40:1,028 40–49:1,473 50–59:1,069 >60:1,045	Median: 6.63	Nasopharyngeal (4,615)	N/P	RT: 59/3594 Non-RT:20/1021	RT: 0.035 No-RT: 0.017	Overall Adjusted RR **1.90** (1.53–2.35)^▴^^$^^∧^^!^ (adjusted for sex, age) <40, adjusted RR **5.76** (3.99–8.33) 40–49 adjusted RR **2.30** (1.90–2.78) 50–59 adjusted RR **1.84** (1.57–2.15) >60 adjusted RR **1.06** (0.93–1.20) (Age categories adjusted for gender)
11. El-Fayech et al. (18): France	3,172	Range: <18	Median:26	Brain (447) HL(218) Others including retinoblastoma (2,507)	Brain CA: ARD 22 Gy HL:ARD13 Gy Retinoblastoma: ARD 9 Gy	RT:70/2,202 No-RT: 4/970 (Not published by CA type)	RT: 0.082 No-RT:0.023 Not published by CA type	Adjusted Overall RR**: 6.9** (5.3–8.9)^▴^^∧^ Brain cancer: Adjusted RR: **29.3** (19.6–42.3)^$^ HL: Adjusted RR: **8.3** (3.0–17.9)^$^ Retinoblastoma: N/P (All RRs Adjusted for sex, age)
12. Campen et al. (19): USA	431	Range: <21	Mean: 6.3	Pediatric brain tumor (649)	MD for non-COW radiation:54 (range 54–55.8) Gy MD for COW radiation:55.8 (range 54–59.4) Gy	RT:13/263 Non-RT:1/168	RT: 0.064 No-RT:0.009	Unadjusted RR **8.0** (1.05–62)^▴^^$^^∧^

▴ *Used in the meta analysis of overall RR*.

$ *Used in the subgroup meta-analysis of CA type*.

! *Used in the subgroup meta-analysis of age*.

∧ *Used in the subgroup meta-analysis of region*.

*For the definition of “stroke,” see text. RR, Relative risk; TD, total dose; MD, median dose; ARD, average radiation dose; RT, radiotherapy; Yr, years; COW, Circle of Willis; Gy, gray; N/P, not published; HL, Hodgkin Lymphoma; NHL, Non Hodgkin Lymphoma; CA, cancer; PNET, primitive neuroectodermal tumor*.

### Statistical Analysis

RR (95% CI) was the comparator for this meta-analysis and HR (95% CI) was considered equivalent to RR. STATA (version 12.0) was used to conduct the meta-analysis (see online available: https://www.stata.com/). Subgroup meta-analyses were done according to a particular variable (such as cancer type) when there were at least two studies published which provided compatible data with respect to that variable. Statistical heterogeneity was calculated using the *I*^2^ test, and the extent of inconsistency was assessed using the *I*^2^ statistic. In general, an *I*^2^ value ≥50% was considered as evidence of heterogeneity, and a random-effects model was selected for the meta-analysis. An *I*^2^ value <50% was considered as evidence of low heterogeneity, and a fix-effects model was used for the meta-analysis. Each study was given a “% weight” which indicates the degree to which each study influenced the overall effect as displayed in forest plots. The “% weight” is influenced by sample size with larger sample sizes having a bigger impact on the analyses and RR results. Analyses with weights adding to 100% for each of the three CA types, four ages groups, and three regions were conducted. Publication bias of positive results reports was analyzed using Begg's test. A two-tailed *p*-value <0.05 was considered statistically significant. Subgroup analyses were conducted based on stratification by cancer type, age, and country/region.

## Results

### Literature Search and Characteristics of the Included Studies

The initial screening excluded 3,156 studies. After removing duplicates, 962 studies remained. Additional screening determined that 303 were conference abstracts without full text or case reports, 123 were not original research, 247 did not provide relative data, 160 were repeat publications of an already included study, and 117 were not studies of humans. A total of 12 studies that fit all inclusion and exclusion criteria were included in the meta-analyses (6, 9–19).

**Figure 1:**
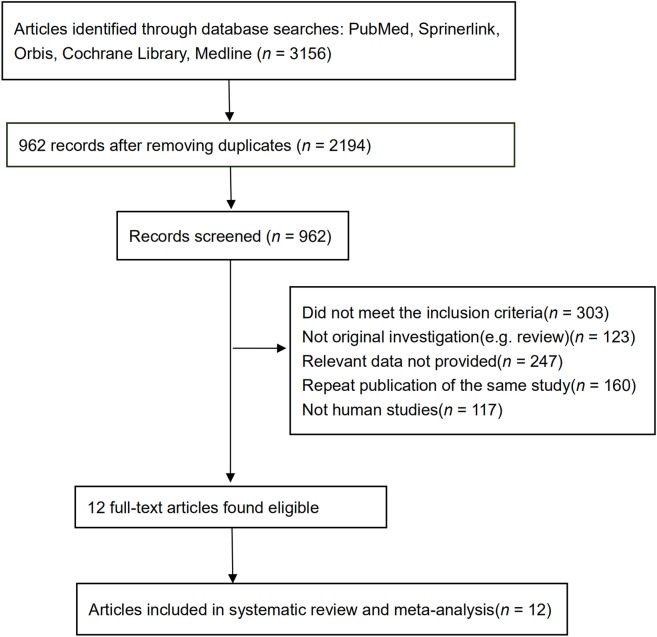
The search process and number of studies included/excluded at each step.

Figure 1 depicts the search process and lists the number of studies included/excluded at each step. All the included studies were retrospective studies. No prospective studies were identified in the searching processing. Table 1 lists the characteristic profile of each study, including the author name, study year, number of participants, baseline age of the participants, number of stroke cases, information on radiation dose, the unadjusted RR, and the relative risk after adjustment for factors such as age and gender (if published). Among these 12 studies, 57,881 total cancer patients were enrolled. The patients underwent radiotherapy with the total dose range from 13 Gray (Gy, is the unit of absorbed dose, 1 Gy represents 1 joule energy absorbed per kilogram of material) to 80 Gy, which was administrated fractionally by 1.5–2 Gy each time. Mean baseline age across studies was estimated to be 51.4 years.

We identified six cancer types that were separately represented in at least two of the eligible 12 studies: head and neck cancer (9–11), nasopharyngeal cancer (10, 17), lung/thoracic cancer (14, 15), Hodgkin lymphoma (6, 13, 15, 18), nasopharyngeal cancer (10, 17), and brain cancer (12, 13, 18, 19). Nasopharyngeal cancer and brain cancer were combined to head and neck cancer since the cancer sites located at the head position. Thus, three types CA can be used for separate subgroup analyses: head/neck/brain/nasopharyngeal cancer (9–13, 17–19), lung/thoracic cancer (14, 15), Hodgkin lymphoma (6, 13, 15, 18). Separately counted cancer types that were represented in only one of the 12 eligible study were oral, testicular, leukemia, non-differentiated lymphoma, non-Hodgkin's lymphoma, neuroblastoma, soft tissue sarcoma, kidney, bone, and breast (10, 13, 15, 16). Subgroup meta-analyses could not be done for these, or other cancer types, due to insufficient published data.

Two of the 12 eligible studies stratified stroke rates according to treatment by baseline patient age (10, 17). However, the categories of baseline patient age differed and only one aged stratified patients according to our predefined categories (<40, 40–49, 50–59, >60 years) (10, 17). Therefore, we matched baseline age from the second study [which categorized patients as aged less than or older than 55 years (10, 17)] as closely as possible in order to perform a subgroup meta-analysis by baseline patient age. Four of the 12 eligible studies were conducted in the USA (11, 13, 15, 19), four in Europe (6, 12, 16, 18), three in Asia (10, 14, 17), and one in Canada (9). The definitions of stroke, when given, varied among the included studies (10, 13, 16, 17, 19). In seven studies the definition of stroke was not given (6, 9, 11, 12, 14, 15, 18). Radiation dose was not used to conduct subgroup meta-analysis because some study authors reported mean and others reported maximal doses, which were not meaningfully comparable.

### Subsequent Stroke Incidence in Cancer Patients Given (Compared to Not Given) Radiotherapy

**Figure 2:**
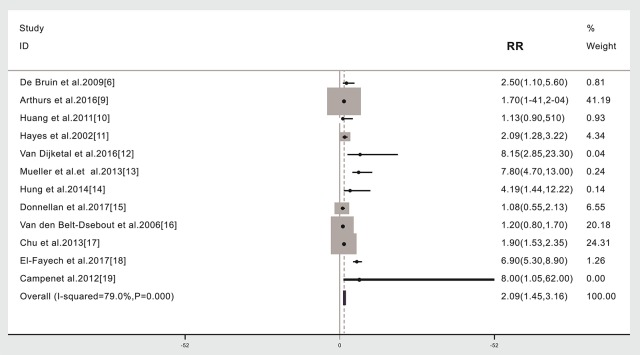
Subsequent stroke incidence in cancer patients given (compared to not given) radiotherapy. The “% weight,” indicates the degree to which each study influenced the overall effect as displayed in forest plots. The “% weight” is influenced by sample size with larger sample sizes having a bigger impact on the analyses and RR results.

The relationship between cancer radiotherapy and subsequent stroke was investigated using data from 12 studies (6, 9–19). Due to significant heterogeneity including different cancer types, different treatment region among the studies, a random model was chosen for the meta-analysis (*I*^2^ = 79.0%, *p* = 0.000). Based on the meta-analysis, the risk of stroke was 2.09 times higher in cancer survivors given radiotherapy compared to those not given radiotherapy (57,881 cases, RR: 2.09, 95% CI: 1.45, 3.16; Figure 2).

### Cancer Type and Subsequent Stroke Incidence in Patients Given (Compared to Not Given) Radiotherapy

**Figure 3:**
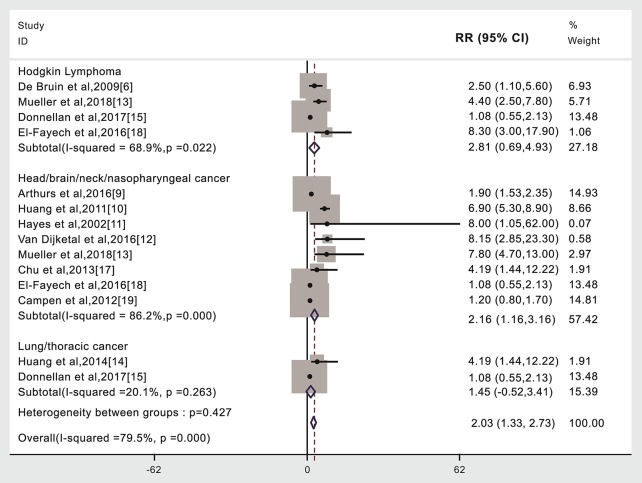
Cancer type and subsequent stroke incidence in patients given (compared to not given) radiotherapy. The “% weight,” indicates the degree to which each study influenced the overall effect as displayed in forest plots. The “% weight” is influenced by sample size with larger sample sizes having a bigger impact on the analyses and RR results.

Stroke risk was higher in patients given radiotherapy if they had Hodgkin's lymphoma (4,438 cases, RR: 2.81, 95% CI: 0.69, 4.93) (6, 13, 15, 18) or head/neck/brain/nasopharyngeal cancer (17,005 cases, RR: 2.16, 95% CI: 1.16, 3.16) (9–13, 17–19). We did not find that the risk of stroke was significantly different in lung/thoracic cancer patients given radiotherapy compared to those not given radiotherapy (732 cases, RR: 1.45, 95% CI: −0.52, 3.41) (14, 15). A subgroup meta-analysis was not possible for the other cancer types due to insufficient published data or the lack of separately published data according to cancer type (Figure 3, Table 1). An analysis with weights adding to 100% for these three CA types subgroups analyses is RR: 2.03 (95% CI: 1.33, 2.73).

### Age at Treatment and Subsequent Stroke Incidence in Patients Given (Compared to Not Given) Radiotherapy

**Figure 4:**
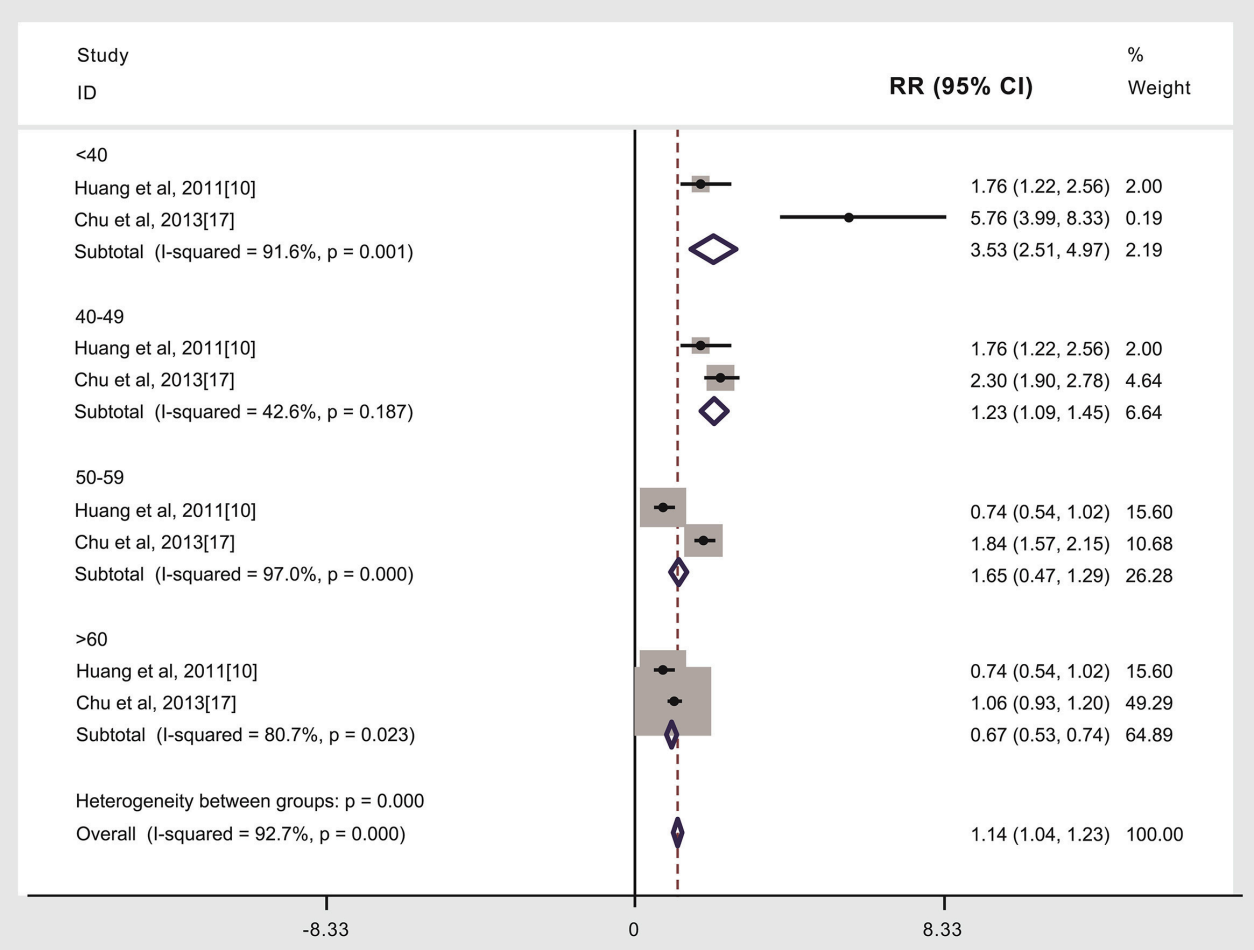
Age at treatment and subsequent stroke incidence in patients given (compared to not given) radiotherapy.The “% weight,” indicates the degree to which each study influenced the overall effect as displayed in forest plots. The “% weight” is influenced by sample size with larger sample sizes having a bigger impact on the analyses and RR results.

Compared to patients not given radiotherapy, stroke risk was higher in patients given radiotherapy if they were <40 years of age when treated (3,073 cases, RR: 3.53, 95% CI: 2.51, 4.97) (10, 17), or 40–49 years of age when treated (3,499 cases, RR: 1.23, 95% CI: 1.09, 1.45) (10, 17), but lower if aged ≥60 years when treated (2,030 cases, RR: 0.67, 95% CI: 0.53, 0.74, Figure 4) (10, 17). We did not find that the risk of stroke was higher in patients given radiotherapy if they were 50–59 years of age when treated (2,588 cases, RR: 1.65, 95%CI: 0.47, 1.29, Figure 4). Other studies weren't included into age subgroup analysis as lacking age subgroups information (see Figure 4, Table 1) (6, 9, 11–16, 18, 19). An analysis with weights adding to 100% for these four ages subgroups analyses is RR: 1.14 (95% CI: 1.04, 1.23).

**Figure 5:**
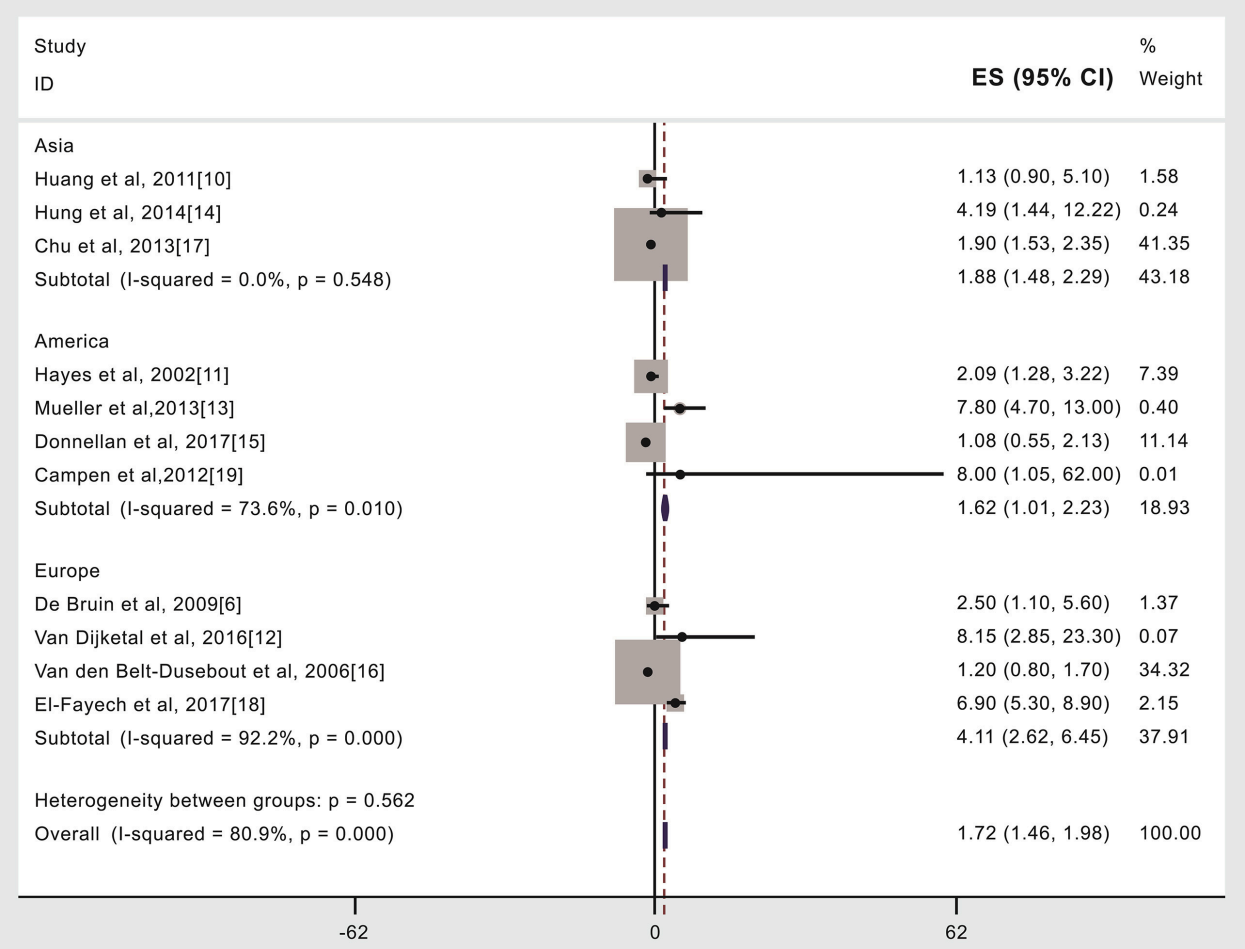
Region or country where treatment occurred and subsequent stroke incidence in patients given (compared to not given) radiotherapy. The “% weight,” indicates the degree to which each study influenced the overall effect as displayed in forest plots. The “% weight” is influenced by sample size with larger sample sizes having a bigger impact on the analyses and RR results.

### Region or Country Where Treatment Occurred and Subsequent Stroke Incidence in Patients Given (Compared to Not Given) Radiotherapy

Compared to patients not given radiotherapy, stroke risk was higher in patients given radiotherapy if they were treated in Asia (15,347 cases, RR: 1.88, 95% CI: 1.48, 2.29) (10, 14, 17) or the United States (19,220 cases, RR: 1.62, 95% CI: 1.01, 2.23) (11, 13, 15, 19) or in Europe (9,245 cases, RR: 4.11, 95% CI: 2.62, 6.45) (6, 12, 16, 18), see Figure 4, Table 2. An analysis with weights adding to 100% for these three region subgroups analyses is RR: 1.72 (95% CI: 1.46, 1.98).

**Table 2:** Summary of studies used in each meta-analysis.

**Meta-analysis**		**No. of studies [references]**	**Total patients for each meta-analysis**	**RRs (95%CI) used in the meta-analyses [reference]**	**RR (95%CI) obtained in the meta-analyses**
Overall meta-analysis	All subjects	12 (6, 9–19)	57,881	**2.5** (1.1–5.6) (6); **1.7** (1.41–2.04) (9); **1.13** (0.9–5.1) (10);**2.09** (1.28–3.22) (11); **8.15** (2.85–23.3) (12); **7.8** (4.7–13.0) (13);**4.19** (1.44–12.22)(14); **1.08** (0.55, 2.13)(15); **1.2** (0.8–1.7) (16); **1.9** (1.53–2.35) (17);**6.9** (5.3–8.9) (18); **8.0** (1.05–62) (19).	**2.09** (1.45, 3.16)
Cancer type for subgroup analysis	Hodgkin lymphoma	4 (6, 13, 15, 18)	4,438	**2.5** (1.1–5.6) (6); **4.4** (2.5–7.8) (13); **1.08** (0.55, 2.13) (15);**8.3** (3.0–17.9) (18)	**2.81** (0.69, 4.93)
	Head/brain/ neck/nasopharyngeal cancer	8 (9–13, 17–19)	17,005	**1.70** (1.41–2.04) (9); **1.13** (0.9–5.1) (10); **2.09** (1.28–3.22) (11);**8.15** (2.85–23.3) (12); **30.1** (17.9–50.8) (13); **1.90** (1.53–2.35) (17);**29.3** (19.6–42.3) (18); **8.0** (1.05–62) (19).	**2.16** (1.16, 3.16)
	Lung/thoracic cancer	2 (14, 15)	732	**4.19** (1.44–12.22) (14); **1.08** (0.55, 2.13) (15)	**1.45** (-0.52, 3.41)
Baseline age	<40	2 (10, 17)	3,073	**1.76** (1.22–2.56) (10); **5.76** (3.99–8.33) (17)	**3.53** (2.51, 4.97)
	40-49	2 (10, 17)	3,499	**1.76** (1.22–2.56) (10); **2.30** (1.90–2.78) (17)	**1.23** (1.09, 1.45)
	50-59	2 (10, 17)	2,588	**0.74** (0.54-1.02)(10); **1.84** (1.57-2.15)(17)	**1.65** (0.47, 1.29)
	>60	2 (10, 17)	2,030	**0.74** (0.54–1.02) (10); **1.06** (0.93–1.20)(17)	**0.67** (CI: 0.53,0.74)
Treatment region	Asia	3 (10, 14, 17)	15,347	**1.13** (0.9–5.1) (10); **4.19** (1.44–12.22) (14); **1.90** (1.53–2.35) (17)	**1.88** (1.48, 2.29)
	America	4 (11, 13, 15, 19)	19,220	**2.09** (1.28–3.22) (11); **7.8** (4.7–13.0) (13); **1.08** (0.55–2.13) (15); **8.0** (1.05–62) (19)	**1.62** (1.01, 2.23)
	Europe	4 (6, 12, 16, 18)	9,245	**2.5** (1.1–5.6) (6); **8.15** (2.85–23.3) (12); **1.2** (0.8–1.7) (16); **6.9** (5.3–8.9) (18)	**4.11** (2.62, 6.45)

*RR and 95% CIs for HL, Head/neck/nasopharyngeal CA, age <40, age 50–59 and Asia, USA, Europe are different to the respective subgroup analyses in Figures 3–5. Please use only the correct results throughout, including in the main text*.

### Publication Bias

Publication bias occurs when significant positive results are more likely to be published than negative or inconclusive results. To explore whether there was publication bias in this study such as publishing positive results rather than negative correlations of radiotherapy with increased subsequent stroke risk, Begg's test was performed and if *p* < 0.05, it is considered that publication bias exists (20). The result of *p*-value from Begg's test was 0.174, which indicates that it was unlikely that this form of publication bias was present in this meta-analysis.

## Discussion

Our overall meta-analysis involving 57,881 total patients indicated that cancer patients who receive radiotherapy have a 2.09 times greater risk of subsequent stroke than cancer patients who do not receive radiotherapy. In addition, from our subgroup meta-analyses, compared to patients not given radiotherapy, stroke risk was higher in patients given radiotherapy if they had Hodgkin's lymphoma (RR: 2.81, 95% CI: 0.69, 4.93) (6, 13, 15, 18) or head/neck/brain/nasopharyngeal cancer (RR: 2.16, 95% CI: 1.16, 3.16) (9–13, 17–19), or if younger than 40 years when treated (RR: 3.53, 95% CI: 2.51, 4.97) or aged 40–49 years when treated (RR: 1.23, 95% CI: 1.09, 1.45) and if they were treated in Asia (RR: 1.88, 95% CI: 1.48, 2.29) or the United States (RR: 1.62, 95% CI: 1.01, 2.23) or in Europe (RR: 4.11, 95% CI: 2.62, 6.45). However, we did not find that the risk of stroke was significantly different in lung/thoracic cancer patients given radiotherapy compared to those not given radiotherapy (732 cases, RR: 1.45, 95% CI: −0.52, 3.41) (14, 15). We also found that the RR of stroke was lower in cancer patients aged >60 years when treated. Therefore, overall and in most subgroup analyses, our results are consistent with previous studies showing an increased future stroke risk associated with radiotherapy (10). However, the reasons for this apparent correlation of higher stroke rate with radiotherapy exposure are not clear from our study. For example, it is possible that sicker patients tend to be given radiotherapy in favor of chemotherapy. If the correlation we have found is real, then it may be patient factors, and not just treatment or investigational factors, are responsible.

Our study methods only compensate for some of the limitations in past studies and so our results require cautious interpretation. However, our study is the best analysis of past studies we are aware of. Our work in trying to make sense of the past literature demonstrates that a systematic and more detailed understanding of the complications of radiotherapy, and how to prevent them, is urgently required. It is the responsibility of all those who provide cancer services to ensure that key information regarding cancer type and load, comorbidities, treatment chosen and short and long-term outcomes of all their patients are documented and analyzed using quality registries. Further, randomized trials may be required to better compare risks according to different treatments of similar cancer patients.

Our subgroup meta-analyses, in particular, require cautious interpretation. We didn't find a higher stroke rate for lung/thoracic cancer patients perhaps because of the relatively small sample size. In addition, we found that stroke rate following ionizing radiation was inversely proportional to patient age until age 60 years. This may be something to do with normal tissue development and/or latency between radiation exposure and stroke. Further, stroke becomes more common with increasing age and larger sample sizes may be required to show differences in stroke risk associated with radiotherapy treatment in older compared to younger persons. Possible factors influencing the stroke risk associated with radiotherapy in different countries or regions are the way radiotherapy is used, the degree of diligence in following patients up after cancer therapy and diagnostic methods, including the definitions of stroke (21).

In addition, we could not test the influence of radiotherapy dose due to some reporting mean and others reporting maximal dose. Other factors that are likely to influence the risk of stroke or other complications associated with radiotherapy include body part irradiated, radiotherapy spacing (7) and the presence or absence of other stroke risk factors. We could not test for such associations because of insufficient published information.

We used relative risk in our meta-analyses as the main indicator of an association between radiotherapy exposure and subsequent stroke incidence. However, the average annual stroke rate is the measure of the absolute effect size. Among our 12 eligible studies, where this information was published, the mean average annual stroke rate was higher for radiotherapy treated patients (mean, 0.413, range: 0.035–1.6) than that for non-radiotherapy treated patients (mean, 0.277, range: 0.009–0.968). The average annual stroke rate is cumulative over the period of observation in each study and it is the most important measure for efforts and studies to improve patient outcomes. In our future investigation, we will use average annual stroke rate and prepare a follow-up paper. It is suggested that all future studies of radiotherapy effects incorporate sufficient data to calculate the average annual rate of all outcomes of interest.

Mechanisms underlying stroke risk as a result of radiation exposure have been addressed in previous radiobiology studies. An increasing number of studies have indicated that post-irradiation damage to the arteries and heart is one of the most common undesirable effects of radiotherapy in cancer patients (22). These arterial changes may lead to late adverse effects of radiotherapy such as strokes or ischemic attacks (23). Moreover, morphological acute changes such as carotid artery blowout, pseudoaneurysm, and long-term changes such as increased intima-media thickening enhance the stroke risk. Notably, ionizing radiation has been indicated to damage the structures of heart and large arteries, resulting in accelerated atherosclerosis and myocardial fibrosis and eventually, leading to ischemic stroke (24). The incidence of carotid stenosis ranged from 18 to 38% in cancer patients with radiotherapy vs. from 0 to 9.2% among cancer patients not given radiotherapy (25). For the heart, radiotherapy produces radiation-induced damage to the myocardium caused by damage to the microvasculature, leading to focal ischemia, interstitial fibrosis and capillary loss (26). Small- and medium-sized blood vessels of brain may be impaired by ionizing radiation, inducing an inflammatory reaction in vessel walls (27).

Multiple forms of biochemical damage have been reported happening after secretion of inflammation factors under radiation exposure such as interleukins, intercellular adhesion molecule 1 (ICAM-1) and vascular cell adhesion molecule 1 (VCAM-1) (28). Additionally, radiation exposure increased adhesiveness of aortic endothelial cells was reported in chemokine-dependent signaling from endothelial cells to leukocytes; such a change in the adhesiveness of vascular endothelial cells could result in the pro-atherogenic accumulation of leukocytes (29). Ionizing radiation has also been shown to have an effect on the likelihood of cerebral amyloid angiopathy (CAA) (30). Adisintegrinand metalloprotease 10 (ADAM10) protease competes with beta-site amyloid precursor protein cleaving enzyme 1 (BACE-1) for amyloid precursor protein (APP). Stroke has a strong association with amyloid angiopathy (31). There is compelling evidence that ionizing radiation can damage DNA double-strand break repair, which is critical for the maintenance of vessel wall genome stability (32–35).

As the global incidence of cancer increases (36), more cancer patients are treated with radiation and have to contend with the subsequent risk of stroke. Prevention strategies to reduce the risk of stroke, and other complications associated with cancer treatment, include reducing the patient's time in accelerator room, wearing personal protective equipment and attention on the ventilation of acceleration room (37) and may include being more selective about using radiotherapy in younger patients or according to cancer type. However, much clearer understanding of the risk of treatment complications (such as stroke) and benefits according to diagnosis is essential to apply preventive strategies effectively. Further, this information needs to be current and locally applicable. The information required can only be supplied if all cancer service providers have all their patients followed up using quality clinical registries. Providing cancer services should be conditional upon use of such registries and systematic efforts to optimize patient outcomes.

Furthermore, reporting standards for studies of health service outcomes, such as radiotherapy and subsequent stroke risk, need improvement. Minimal standards for publishing such studies are scientifically sound, reproducible, generally applicable, and clinically useful definitions of the condition being treated, the therapeutic interventions used and the outcomes measured. For example, stroke was not defined in 7 of our 12 included studies and it was variably defined in the others. This greatly limits study comparison and assessment of the clinical significance of “stroke” as a complication of radiotherapy. We recommend clinically-based definitions of stroke and transient ischemic attack (TIA) using sub-categorization by brain imaging results, when available (21). In addition, radiation dose used in cancer treatment varies. We recommend they should report the dose they used and compare this with conventional or guideline recommended doses. Specifically, total dose per patient, fraction size, spacing, targeted body part and form of radiation should be published. Meanwhile, it is now clear that previous researchers looking at the risk of stroke/TIA with and without RT have grouped patients mainly according to organ or body part involved, rather than by pathological type. So, for example, head and neck cancer or testicular cancer are very heterogeneous groups. Future studies should be better organized and published by subdividing by pathological types.

## Conclusions

Our meta-analysis indicates an overall doubling of the future risk of stroke in cancer patients given radiotherapy compared to those not given radiotherapy. The statistically significant elevated stroke risk with radiotherapy was also seen with some particular cancer types, with younger baseline patient age and in certain countries/regions where patients were treated. Although our results require cautious interpretation, due to known underlying limitations in the published literature, this is the best analysis of the available research we are aware of. We cannot be sure to the extent that patient, treatment and/or investigational factors are responsible for this apparent correlation of radiotherapy exposure and increased stroke risk. In the very least, our results should raise concern and be used for hypothesis generation. Our results demonstrate an urgent need for more compelling prospective cohort studies to address potential confounding factors and reduce selection bias which obscure the relationship between radiotherapy and stroke incidence. Such studies (whether or not involving randomization) are best done within a system of universal involvement of patients in quality registries. Only this will enable us to provide timely, current and locally relevant data to inform healthcare decisions on an ongoing basis. Additionally, future studies of cancer treatment should be subject to minimal reporting standards including the use of scientifically sound, reproducible, generally applicable and clinically meaningful definitions of cancer types, treatment received, and outcomes of interest (such as stroke).

## Author Contributions

P-KZ conceived and designed the study. RH, FC, GR, and YZ performed eligibility screening and data extraction. RH, GR, SH, and YZ analyzed the data and performed the statistical analysis. P-KZ and RH drafted the initial manuscript. P-KZ critically reviewed and revised the manuscript.

### Conflict of Interest Statement

The authors declare that the research was conducted in the absence of any commercial or financial relationships that could be construed as a potential conflict of interest.
